# Intercalibration of ^40^Ar/^39^Ar laboratories in China, the USA and Russia for Emeishan volcanism and the Guadalupian–Lopingian boundary

**DOI:** 10.1093/nsr/nwz044

**Published:** 2019-03-30

**Authors:** Brian R Jicha, Brad S Singer, Youjuan Li

**Affiliations:** 1 Department of Geoscience, University of Wisconsin-Madison, USA; 2 State Key laboratory of Earthquake Dynamics, Institute of Geology, China Earthquake Administration, China

Precise geochronology is essential for constraining rates of sedimentary, biotic, climatic and magmatic processes over a wide range of timescales throughout Earth's history. Targets for high-precision geochronology include lava flows or minerals from volcanic tuffs that are interbedded within sedimentary strata. High-precision dates from these materials commonly provide temporal anchors, allow disparate stratigraphic records to be correlated with one another and form the basis of the geologic timescale. Obtaining precise and accurate date goes well beyond using an instrument with the capability to acquire such a value. For example, recent breakthroughs in radioisotope geochronology have required community-wide efforts to understand the many systematic and geologic sources of uncertainty and dispersion for a given set of dates in the rock record. In 2003, the EARTHTIME initiative [1] began to promote cooperation and intercalibration among ^40^Ar/^39^Ar and U-Pb geochronology laboratories in the USA and Europe with the aim of producing an accurate, high-resolution, geological time scale. That effort has also aimed to forge collaborations among geochronologists, stratigraphers, climate scientists, geochemists, paleomagnetists and paleontologists.

**Figure 1.**
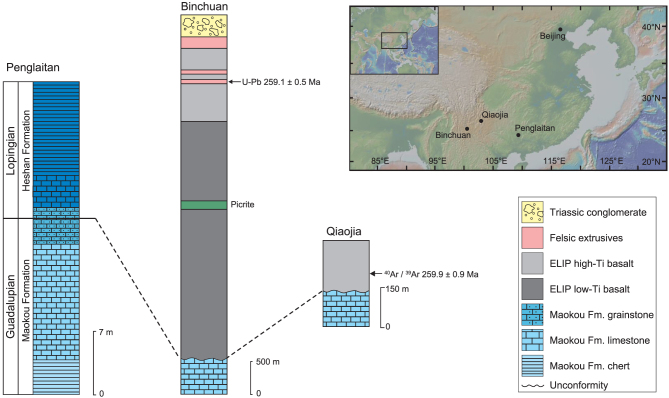
Composite stratigraphic columns at the Penglaitan, Binchuan and Qiaojia sections in southern China after [2,3,7,8]. The map shows the locations of the three sections. The black solid line is the location of the Guadalupian–Lopingian boundary within a grainstone unit at the GSSP in the Penglaitan section. The dashed line is the inferred location of the boundary in the other sections. New ^40^Ar/^39^Ar age based on six experiments in three laboratories is shown with full external uncertainty so that it can be compared to the published U-Pb age.

Recently, EARTHTIME-CN was established to improve geochronological techniques within China and foster intercalibration efforts among laboratories in China, and globally. Another impetus for Earthtime-CN is that there are rich stratigraphic records in China, including 11 Global Boundary Stratotype Section and Points (GSSP), many of which lack high-precision geochronology. One of those GSSPs is the Guadalupian–Lopingian boundary that is located in the Penglaitan section in Laibin County, Guangxi Autonomous Region, South China [2]. The GSSP is defined as the First Appearance Datum of the conodont *Clarkina postbitteri postbitteri* at the base of Bed 6k within a grainstone unit in the Penglaitan section. This point is within a chronomorphocline from *Clarkina postbitteri hongshuiensis* to *C. dukouensis* [2,3]. This section primarily consists of limestone and shales and lacks dateable volcanic material (Fig. 1). The age of the Guadalupian–Lopingian boundary is 259.8 ± 0.4 Ma according to the Geologic Time Scale 2012 [4] or 259.1 ± 0.5 Ma as stated by the International Commission on Stratigraphy [3].

Previous studies (e.g. [5]) have suggested that voluminous volcanism from the Late Permian Emeishan large igneous province (ELIP) may have caused the end-Guadalupian mass extinction at the Guadalupian–Lopingian boundary. Basaltic lavas of the ELIP are divided into high-Ti and low-Ti chemical types. While there is some controversy regarding this classification scheme and the eruptive sequence of the two basalt types, it is generally agreed upon that the high-Ti basalts are preceded by low-Ti basalts, and that the basalts unconformably overlie limestones of the middle Permian Maokou Formation (Fig. 1) [6]. In several sections, the high-Ti basalt is capped by several thin felsic ignimbrites that are inferred to represent the termination of the ELIP [7]. Zircons from one of the ignimbrites in the Binchuan section were dated by the U-Pb chemical abrasion–thermal ionization mass spectrometry (CA–TIMS) method and gave an age of 259.1 ± 0.5 Ma [7].

**Figure 2.**
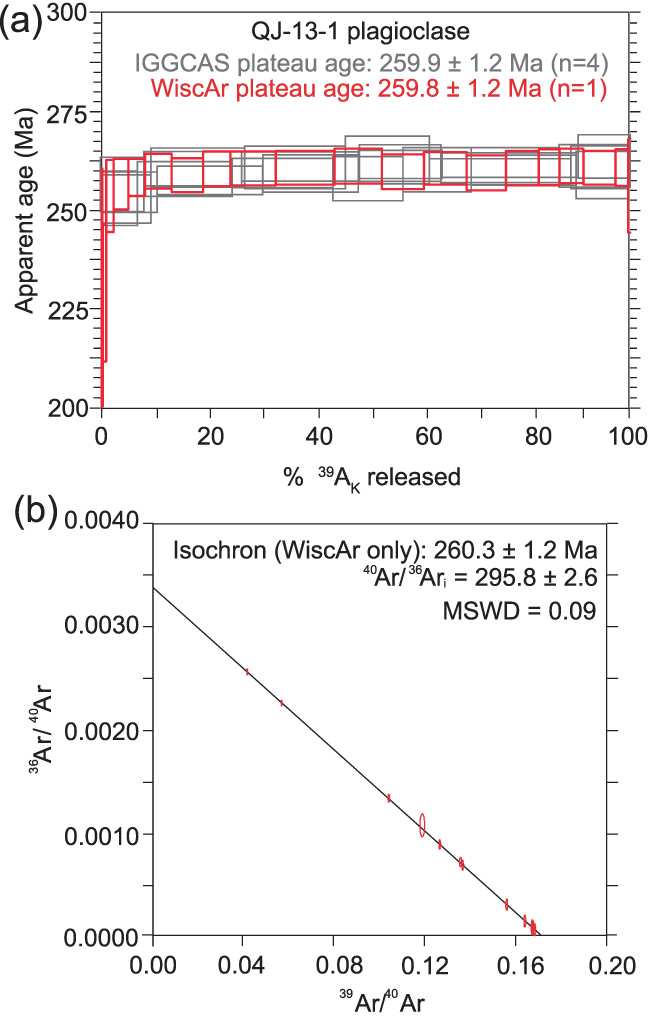
^40^Ar/^39^Ar age spectrum (a) and inverse isochron (b) diagrams for sample QJ-13-1, an ELIP high-Ti basalt from the Qiaojia section [8]. Note the excellent agreement between the Noble Gas lab (IGGCAS, Beijing) and the WiscAr laboratory. The inverse isochron from the WiscAr data indicates no evidence of trapped excess Ar.

Numerous attempts have been made to constrain the age and duration of the ELIP using a variety of chronometers. ^40^Ar/^39^Ar dating of the ELIP basalts has been problematic, as the experiments were primarily conducted on whole-rock samples that yielded disturbed age spectra with ages ranging from ∼252 to 256 Ma, all of which are not in agreement with the 259.1 ± 0.5 Ma U-Pb age from the overlying felsic ignimbrite. Recently, Li *et al.* [8] conducted four ^40^Ar/^39^Ar incremental heating experiments on ∼15 mg aliquots of fresh plagioclase from sample QJ 13-1—a high-Ti basalt from the ELIP at the Qiaojia section located in the southwest of Zhaotong city (the Yunnan province). These data gave a weighted mean age of 259.9 ± 1.2 Ma (2σ analytical uncertainty) [8]. One experiment on a different sample of high-Ti basalt from the Qiaojia section that was analysed at the Center for Geodynamics and Geochronology of the Institute of the Earth's Crust SB RAS in Irkutsk, Russia gave a plateau age of 261.7 ± 3.4 Ma. As part of an Earthtime-CN effort to further assess the accuracy and precision of the ^40^Ar/^39^Ar data generated in the Noble Gas lab at the Institute of Geology and Geophysics, Chinese Academy of Sciences (IGGCAS) in Beijing, sample QJ 13-1 was shared with the WiscAr laboratory at the University of Wisconsin-Madison. A ∼2-mg plagioclase separate was incrementally heated using a 25-W CO_2_ laser and the released gas was analysed using a MAP215-50 mass spectrometer. A plateau comprising 99.1% of the ^39^Ar released gives an age of 259.8 ± 1.2 Ma (2σ) (Fig. 2), which is nearly identical to the age obtained at IGGCAS based on four experiments. All ages discussed herein are calculated relative to a Fish Canyon standard age of 28.201 Ma [9] using the decay constants of [10]. The inverse isochron intercept indicates a trapped ^40^Ar/^36^Ar composition that is within uncertainty of the present-day atmospheric value (Fig. 2) and thus the plateau age is preferred. A weighted mean of the six plateau ages conducted on the high-Ti basalt (Beijing, Irkutsk, Madison) is 259.9 ± 0.8/0.9 Ma (2σ analytical/full external uncertainty). Because there is excellent reproducibility amongst the three laboratories for the QJ 13-1 plagioclase, consideration should be given to making it a new standard for ^40^Ar/^39^Ar geochronology. The new high-precision data suggest that the duration of high-Ti basaltic volcanism may have been as short as 0.8 Ma (259.9–259.1 Ma). In order to properly assess the entire duration of ELIP volcanism, ages must be also obtained for the low-Ti basalts.

Discussion underway between staff at the IGGCAS and Wisconsin laboratories is focused on sample preparation as well as decreasing blanks and improving mass spectrometry with the aim of conducting additional collaborative high-resolution timescale studies within China. This intercalibration effort presented here has highlighted some of the challenges associated with rock-clock calibration, and has reconciled some conflicting ages for the ELIP. Future intercalibration experiments as part of EARTHTIME and EARTHTIME-CN should strive to continue to push the limits of high-precision geochronology and seek unprecedented levels of integration across various scientific methodologies and disciplines.
